# Comprehensive IAC Cross-Reactivity Validation and Stabilized Method Development for Ochratoxin A, B, and C in Complex Coffee and Spice Matrices

**DOI:** 10.3390/foods14234102

**Published:** 2025-11-28

**Authors:** Jiaojiao Xu, Zengxuan Cai, Mengli Wang, Xiaomin Xu, Haitao Shen

**Affiliations:** 1 Zhejiang Provincial Center of Disease Control and Prevention, Hangzhou 310051, China; jjxucdc@163.com (J.X.); zxcai@cdc.zj.cn (Z.C.); xmxu@cdc.zj.cn (X.X.); 2 NHC Specialty Laboratory of Food Safety Risk Assessment and Standard Development, Hangzhou 310051, China

**Keywords:** ochratoxins, ochratoxin A, ochratoxin B, ochratoxin C, coffee, spices, IAC cross-reactivity

## Abstract

Ochratoxins (OTs) pose a major food safety threat, yet analytical methodologies and regulations focus almost exclusively on ochratoxin A (OTA), overlooking the toxic analogues OTB and OTC, especially in complex coffee and spice matrices. The present study addressed this gap by first systematically confirming the high cross-reactivity (>85%) of commercial OTA immunoaffinity columns (IACs) toward OTB and OTC. It was identified that conventional alkaline methanol extraction caused OTC degradation, and subsequently a stable and unified acetonitrile-water (8/2, *v*/*v*) extraction protocol was developed. To overcome severe matrix interference endemic to these foods, a novel 0.5% Tween-20-PBS IAC load and wash procedure was optimized. The resulting method was fully validated in representative roasted coffee and pepper matrices on both HPLC-FLD and UHPLC-MS/MS platforms, demonstrating excellent linearity (r > 0.999), accuracy (mean recovery 82.00–112.51%), and precision (RSD% ≤ 8.81%) across three spiked levels (0.3, 5, 10 µg/kg). While UHPLC-MS/MS achieved higher sensitivity (LOQs 0.1 µg/kg) than that of HPLC-FLD (LOQs 0.3 µg/kg), with isotope internal standards essential for correcting significant matrix effects. Application to forty commercial coffee and spice samples (19 coffee, 21 spice) revealed OTA contamination in 47.5% of products (up to 3.46 µg/kg) and co-occurrence of OTA/OTB in 3 of 8 cumin samples. This work establishes the first comprehensively validated IAC-based method for multi-OTs in complex foods, facilitating an urgently needed, robust tool for comprehensive risk assessment.

## 1. Introduction

The global food commodities of coffee and spices encompass an immense spectrum of products vital to international trade and culinary traditions [1,2]. Due to their cultivation in often warm and humid climates and the extended processes of global transportation and storage, these commodities are highly susceptible to contamination by toxigenic fungi like Aspergillus and Penicillium. The resulting mycotoxins, particularly ochratoxins, pose a significant and persistent threat to human health due to their nephrotoxic, carcinogenic, and immunotoxic properties [3].

The ochratoxin family is primarily composed of ochratoxin A (OTA), ochratoxin B (OTB), and ochratoxin C (OTC). OTA is the most prevalent and toxic member, exhibiting a formidable range of adverse health effects, including potent nephrotoxicity, hepatotoxicity, teratogenicity, immunotoxicity, neurotoxicity, and mutagenicity [4,5]. Owing to its well-documented toxicity, the International Agency for Research on Cancer (IARC) has classified OTA as a Group 2B possible human carcinogen. While studied less extensively, its structural analogues are far from benign. OTB, the non-chlorinated form of OTA, and OTC, its ethyl ester, also demonstrate significant toxicity, including nephrotoxic, hepatotoxic, and immunotoxic effects [6,7]. Although OTB is generally considered less toxic than OTA, its presence contributes to the overall toxicological burden. Furthermore, studies have demonstrated that these analogues can interconvert even at very low concentrations, with OTB and OTC potentially being transformed into the more toxic OTA [6,7]. This dynamic interplay is compounded by the potential for additive or synergistic toxic effects when consumed as a mixture, posing a greater and more complex threat to human and animal health than any single compound alone.

Humans and animals are always exposed to mixtures of mycotoxins rather than to individual ochratoxins [8,9,10]. OTA and OTB often co-occur in foods, as reported in red wine [11,12], coffee [13], and cereals [14,15]. To minimize public exposure, European Commission Regulation 2023/915 stipulates a maximum permissible residue limit (MRL) of 3 µg/kg for roasted coffee and 15–20 µg/kg for certain spices [16], while China’s National Food Safety Standard (GB 2761-2017) sets an MRL of 10 µg/kg for coffee [17]. These limits serve as critical benchmarks for ensuring food safety and risk evaluation. However, a critical regulatory gap exists, as maximum limits for OTB and OTC have not been established in most jurisdictions. This absence is partly due to a historical lack of comprehensive occurrence data, underscoring the urgent need for robust analytical methods that can generate reliable data to inform future risk assessments and the potential establishment of regulatory standards for these compounds.

While various analytical techniques for OTA exist, liquid chromatography (LC) methods are the most prevalent and effective for the simultaneous quantification of multiple components [18,19,20,21,22]. HPLC coupled with fluorescence detection (HPLC-FLD) or mass spectrometry (UHPLC-MS/MS) remains the routine method for ochratoxin A (OTA) analysis in food, offering superior sensitivity. HPLC-FLD, in particular, serves as the primary technique in international standards (AOAC, EN, ISO) and has been applied to matrices like dried fruits, beer, coffee, milk, and wine. Sample preparation is critical, especially for complex matrices like roasted coffee and spices, which pose significant analytical challenges. Immunoaffinity columns (IACs) are favoured for their efficient cleanup and high binding capacity, leveraging antigen–antibody specificity for superior purification and enrichment of target compounds [21,23]. However, a critical gap exists: commercial IACs are typically validated for OTA. Systematic evaluations of their cross-reactivity and binding efficiency for OTB and OTC are largely absent, limiting their validated use for simultaneous multi-ochratoxin detection. This analytical gap is mirrored in regulatory frameworks. International standard methods focus almost exclusively on OTA, driven by historical constraints and unverified cleanup performance for its analogues. This OTA-centric approach has resulted in significant data gaps regarding OTB and OTC exposure risks. While several published studies on OTs detection exist [11,14,15,24], they primarily address simpler matrices like wines and grains. Reports on complex matrices such as spices and coffee are sparse, and robust, unified methods applicable across diverse matrices are even rarer. Furthermore, disparate extraction solvents used in existing protocols risk inconsistent recovery efficiencies due to polarity differences among the OTs.

This study aimed to address these identified gaps. First, the binding specificity and cross-reactivity of commercial IACs for OTB and OTC were systematically evaluated. Based on these findings, a comprehensive IAC cleanup method was subsequently optimized and fully validated for the simultaneous determination of OTA, OTB, and OTC in coffee and spices, utilizing two detection modes (HPLC-FLD and UHPLC-MS/MS). This single, unified pretreatment protocol, applicable to diverse food categories, provides a robust and versatile framework for broader OT monitoring. To our knowledge, this study represents the first comprehensive optimization and validation of an IAC-based method for multi-OTs analysis in such complex food matrices.

## 2. Materials and Methods

### 2.1. Chemicals and Reagents

Standard solutions of OTA, OTB, and OTC (10 μg/mL, purities ≥ 99%), and the isotopes ^13^C-OTA, ^13^C-OTB, and ^13^C-OTC (10 μg/mL, purities ≥ 98%) in acetonitrile were purchased from Pribolab Pte. Ltd. (Qingdao, China). Methanol (MeOH), acetonitrile (ACN), acetic acid, and formic acid were HPLC grade and purchased from Merck (Merck, Germany). Phosphate-buffered saline (PBS, pH 7~7.4) and Tween-20 were purchased from Sinopharm Chemical Reagent Co., Ltd. (Shanghai, China). Ultrapure water was obtained by using a Milli-Q Millipore system (Bedford, MA, USA). OTA-Clean IACs were supplied from Meizheng Bio-tech Co., Ltd. (Rizhao, China).

### 2.2. Instrumental

HPLC analysis was performed on an ACQUITY HPLC™ system coupled to fluorescence detection (Waters, Singapore). Separation was achieved on a CNW Athena C18-WP column (150 mm × 4.6 mm i.d., 5 µm particle size, Anpel Laboratory Technologies (Shanghai) Inc., Shanghai, China), with a 50 µL injection volume. The column temperature was maintained at 40 °C. Mobile phase solvent A was a 1% acetic acid solution, and solvent B was MEOH/ACN (1/1, volume ratio). Mobile phase B was increased from 65 to 80% in 0–10 min and was then held constant at 100% during 11–13 min. The system was then re-equilibrated during 13.5–20 min in the initial mobile phase composition. Fluorescence detection parameters were selected at λ_ex_/λ_em_ = 333 nm/460 nm.

UHPLC-MS/MS analysis was carried out on a Sciex QTRAP 6500+ (Sciex, Singapore) mass spectrometer, which is coupled to a Shimadzu 30A UHPLC system (Shimadzu Technologies China Ltd., Beijing, China). The injection volume (5 μL) was kept constant throughout the analysis. Mass spectrometer operating conditions were as follows: Spray voltage- 5500 V, curtain gas- 30 psi, temperature-500 °C, gas 1- 30 psi, gas 2- 50 psi, and entrance potential (EP)- 10 V. Other parameters, including declustering potential (DP), exit potential (CXP), and collision energy (CE), were optimized based on the compound and listed in Table 1. The UHPLC system operating conditions were as follows: Mobile phase solvent A was a 0.1% formic acid solution, and solvent B was ACN (0.1% formic acid). A BEH C18 column (100 mm × 2.1 mm, 1.7 μm, Waters, Inc.) was used for the separation of ochratoxins under a 40 °C column temperature. An optimized gradient to achieve maximum separation (0 to 6 min—40%B, 6 to 8 min—40%B to 70% B, 8 to 9 min—70%B to 100%B, 9 to 11 min—100%B, 11 to 11.5 min—100%B to 40%B, 11.5 to 13 min—40%B) at 0.3 mL/min flow rate was used for analysis.

### 2.3. Coffee and Spice Samples

Coffee and spice samples were obtained from several supermarkets and online shops in Zhejiang Province. The following types of coffee and spice samples were selected: black coffee, instant coffee, roasted coffee, Sichuan pepper, cumin, and white pepper. All of the forty samples were completely ground and homogenized with a blender jar and were kept in darkness at room temperature until the analysis.

### 2.4. Optimization Parameters for Ochratoxin Extraction and Clean-Up

The optimization of extraction and clean-up was carried out for ochratoxins (OTA, OTB, and OTC) by representative sample matrices, including roasted coffee and Sichuan pepper.

#### 2.4.1. Optimization of Extraction

Currently, effective standard methods and published literature for the determination of OTs in coffee and spice samples utilize various extraction solution systems [14,23,25,26,27,28], including alkaline water, MeOH-water, and aqueous MeOH-sodium bicarbonate solutions. Significant discrepancies in solvent composition exist between these methods, posing potential risks to the consistency of results and compliance evaluations for import/export inspections.

Given that the ACN-water system is a highly common extraction solvent in mycotoxin analysis, a systematic investigation was conducted to compare different ratios of ACN-water, MeOH-water, aqueous ACN-sodium bicarbonate (NaHCO_3_), and aqueous MeOH-NaHCO_3_. The stability of the target OTs (OTA, OTB, and OTC) in these systems and their extraction efficiency from real samples were evaluated, respectively.

First, the stability of individual OTA, OTB, and OTC standard solutions (1 μg/mL) was assessed at room temperature in ACN-3% NaHCO_3_ (5/5, *v*/*v*) and MeOH-3% NaHCO_3_ (5/5, *v*/*v*), with concentrations analyzed after 0, 1, 3, and 7 days of storage.

Subsequently, the representative matrices were used to evaluate the extraction efficiency of OTA, OTB, and OTC. Due to significant precipitation of NaHCO_3_ observed in ACN-1% NaHCO_3_ (8/2, *v*/*v*), ACN-3% NaHCO_3_ (7/3, *v*/*v*), and ACN-3% NaHCO_3_ (6/4, *v*/*v*) systems, these combinations were excluded. The extraction solutions systematically investigated therefore included MeOH-water (5/5, *v*/*v*), ACN-water (5/5, *v*/*v*), ACN-1% NaHCO_3_ (5/5, *v*/*v*), ACN-3% NaHCO_3_ (5/5, *v*/*v*), MeOH-water (6/4, *v*/*v*), ACN-water (6/4, *v*/*v*), ACN-1% NaHCO_3_ (6/4, *v*/*v*), MeOH-water (7/3, *v*/*v*), ACN-water (7/3, *v*/*v*), ACN-1% NaHCO_3_ (7/3, *v*/*v*), MeOH-water (8/2, *v*/*v*), and ACN-water (8/2, *v*/*v*).

To directly reflect extraction efficiency, a dilute-and-shoot UHPLC-MS/MS method was employed. The procedure was as follows: 5 g of the sample was weighed, spiked with 100 ng of OTs standard, vortex-mixed, and incubated overnight. Then, 25 mL of extraction solvent was added, followed by vortex extraction for 30 min. After centrifugation at 6000 rpm for 10 min, 0.5 mL of supernatant was mixed with 1.5 mL of ACN-water (5/5, *v*/*v*) and filtered through a 0.22 μm membrane. Finally, a 98 μL aliquot of filtrate was combined with 2 μL of mixed isotope internal standard (100 ng/mL) and analyzed by UHPLC-MS/MS. Extraction recoveries were calculated using the internal standard method.

#### 2.4.2. Optimization of IAC Clean-Up

Immunoaffinity columns (IACs) possess a defined tolerance for the organic solvent ratio in the loading solution. Generally, IAC tolerance is within ≤8% for ACN and ≤20% for MeOH, and the loading solution pH should be maintained between 7 and 8. A conventional IAC loading system was employed, wherein the ACN-water extract was diluted with PBS to reduce the ACN concentration to ≤8% before loading. During the experimental process, it was observed that extracts from matrices such as Sichuan pepper and coffee became turbid upon dilution with PBS, frequently causing IAC clogging during the loading step. However, diluting the extract with PBS buffer containing Tween-20 (PBS-T) was found to reduce the turbidity of the loading solution. Simultaneously, this approach prevented coloured substances from the coffee and pepper matrices from adsorbing onto the IAC packing material, thus achieving a superior impurity removal effect [26].

In the loading step, a systematic comparison was conducted to evaluate the cleanup efficiency of PBS-T buffers containing different percentages of Tween-20 (0%, 0.1%, 0.5%, 1%, 2%) against interfering substances, such as coloured materials, in representative sample matrices. A 4 mL aliquot of the extract was taken and spiked with 100 ng of a mixed OTs standard solution. These spiked aliquots were then diluted to 50 mL using PBS-T solutions with varying Tween-20 concentrations, followed by IAC loading, washing, and elution. The cleanup efficiency and spiked recovery rates were subsequently compared using HPLC-FLD analysis.

In the washing step, the effect of adding a PBS-T wash step, using the same Tween-20 concentration as the dilution buffer, was also investigated. Using the representative matrices, a 4 mL extract aliquot (containing 100 ng OTs) was diluted to 50 mL with PBS-T solution and loaded. The cartridge was then washed sequentially with 10 mL of PBS-T solution and 10 mL of PBS. The OTs were eluted with 2 mL of 2% acetic acid in MeOH. The final eluate was analyzed by HPLC-FLD, and the recovery rates were calculated.

### 2.5. Sample Pretreatment

The sample pretreatment process is described as follows, and the workflow is illustrated in Figure S1. Five grams of homogenized coffee or spice samples were weighed into a 50 mL centrifuge tube (for UHPLC-MS/MS detection, 50 μL of 100 ng/mL mixed isotope standard solution was added before extraction). The acetonitrile-water (8/2, *v*/*v*) extraction solvent was specifically chosen to maintain the stability of OTC by avoiding the alkaline conditions (e.g., NaHCO_3_ addition) that promote ester hydrolysis and degradation. After adding 20 mL of ACN-water (8/2, *v*/*v*) extraction solvent, the centrifuge tube was shaken on a multi-tube vortexer for 20 min and then centrifuged at 10,000 rpm for 5 min.

An amount of 4 mL of the supernatant was diluted in 45 mL of PBS-T solution. Confirmed the use of PBS (pH 7–7.4) for the loading and washing steps, ensuring a pH-stable environment for OTC. After mixing and centrifuging at 10,000 rpm for 3 min, all of the supernatant was slowly passed (vacuum-free) through the IAC. It was washed with 10 mL PBS-T and 10 mL PBS, respectively, before the elution with 2 mL of 2% acetic acid in MeOH. Finally, the eluate was evaporated to dryness under a gentle stream of nitrogen at 40 °C. The dried eluate was dissolved in 0.5 mL of the initial mobile phase for the analyte.

### 2.6. Matrix Effect Evaluation of LC-MS/MS

To assess the matrix effect (ME) across various types of coffee and spices, matrix-matched isotope-standard (ISTD) solutions were established using representative samples, while the ISTD solution was prepared in solvent. Five matrix samples, including roasted coffee, instant coffee, Sichuan pepper, cumin, and white pepper, were selected and pretreated as described in Section 2.5 to obtain a matrix solution. The mixed ISTD was added, and the matrix-matched solutions were configured at a concentration of 0.5 ng/mL. The ISTD solution in the solvent was diluted with the initial mobile phase to the same concentration levels. ME was evaluated by calculation equal to the following: [29]: *ME*% = *Area*_matrix-matched ISTD_/*Area*_solvent-matched ISTD_ × 100.

### 2.7. Method Validation and Comparison

The developed method was validated in-house by two representative matrix samples (including roasted coffee and Sichuan pepper) in terms of recovery, linearity, limit of detection (LOD), and limit of quantification (LOQ). Linearity was assessed using seven OT concentrations (0.5, 1.0, 2.0, 5.0, 10.0, 20.0, and 50.0 ng/mL for HPLC-FLD; 0.1, 0.5, 1.0, 2.0, 5.0, 10.0, and 20.0 ng/mL for UHPLC-MS/MS). LODs and LOQs were calculated as the minimum OT concentrations producing chromatographic peak areas with signal-to-noise (S/N) ratios of 3 and 10, respectively. Recovery was evaluated by spiking OT’s standard solution at three different concentrations (0.3, 5, and 10 μg/kg) in the blank representative samples (roasted coffee and Sichuan pepper) in six replicates. Spiked samples were left overnight at room temperature to allow the added standard solution to evaporate. Precision and accuracy were evaluated from recovery experiments.

Method comparison was performed using FAPAS quality control materials (FAPAS17232, Coffee) and 2022 proficiency testing samples (Mixed spices) in six replicates. As only OTA results are specified in available quality control materials and proficiency testing schemes, the comparative analysis was limited to OTA.

## 3. Results and Discussion

### 3.1. Optimization of Extractive Solvents on Ochratoxin Recovery

To develop a robust extraction method for the simultaneous determination of OTA, OTB, and OTC, this study first addressed the inconsistencies of current standard methods and published studies [14,23,25,26,27,28]. Existing protocols (e.g., EN 17250:2020 [30]; EN 14132:2009 [31]) employ varied solvents, notably MeOH-bicarbonate solutions, which were primarily validated for OTA. This study evaluated the stability of all three analogues in these systems.

**Figure 1:**
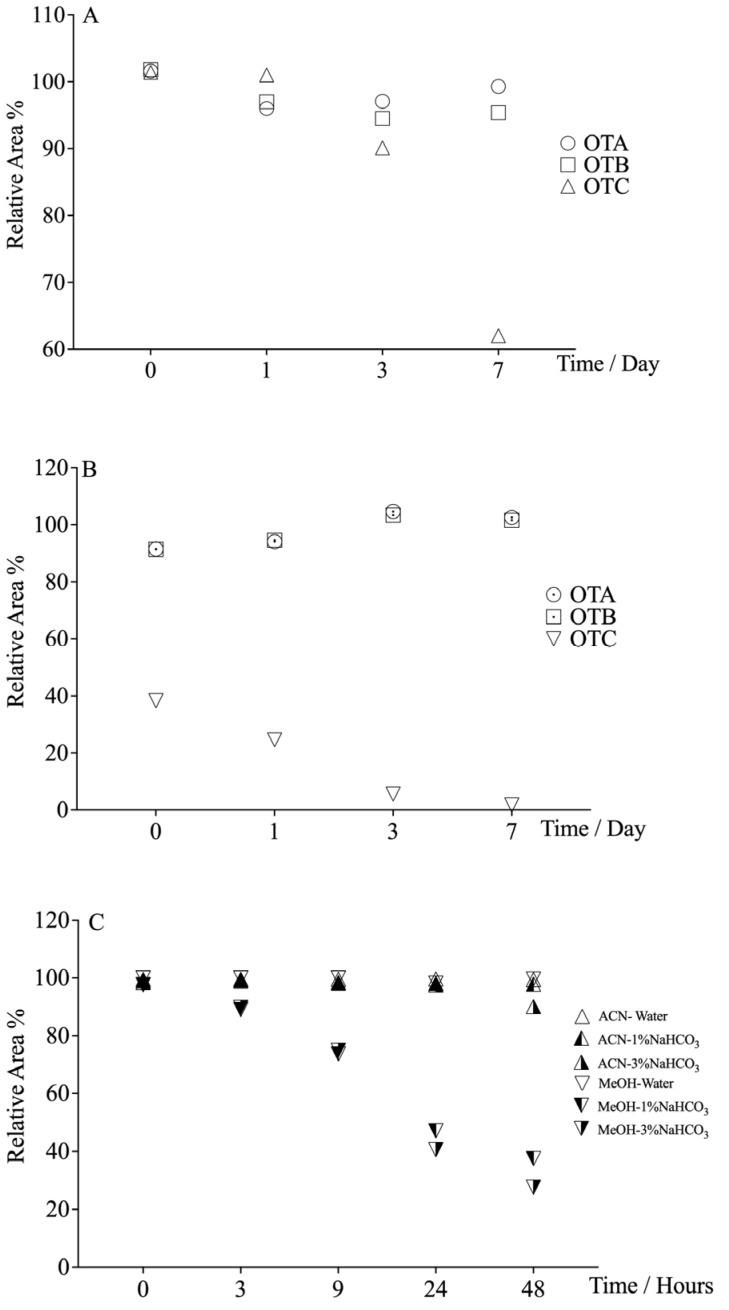
Stability of ochratoxins in organic solution with sodium bicarbonate (5/5, *v*/*v*). ((**A**): OTs in ACN-3% NaHCO_3_ solvent; (**B**): OTs in MeOH-3% NaHCO_3_ solvent; (**C**): OTC in ACN or MeOH with bicarbonate solution).

As shown in Figure 1A,B, OTA and OTB remained stable across tested systems, whereas OTC demonstrated significant degradation in both ACN and MeOH bicarbonate solvent systems, attributable to ester hydrolysis under alkaline conditions. Notably, the initial OTC concentration in MeOH-bicarbonate solvent was only 50% of that in the ACN-based counterpart (Figure 1B), potentially related to analysis timing. And OTC loss was accelerated in MeOH-bicarbonate solvent (declining 70% in 24 h) versus acetonitrile counterparts, likely due to methanol’s nucleophilic facilitation of transesterification. Thus, further investigation of OTC stability in the MeOH-bicarbonate system for a shorter time was evaluated. As shown in Figure 1C, there were negligible differences in OTC stability between 1% and 3% alkaline concentrations. And it exhibited rapid degradation in MeOH bicarbonate medium: approximately 10% depletion at 3 h, 30% at 9 h, and 70% at 48 h. These kinetics underscore acetonitrile’s superiority for preserving OTC integrity during routine handling.

**Figure 2:**
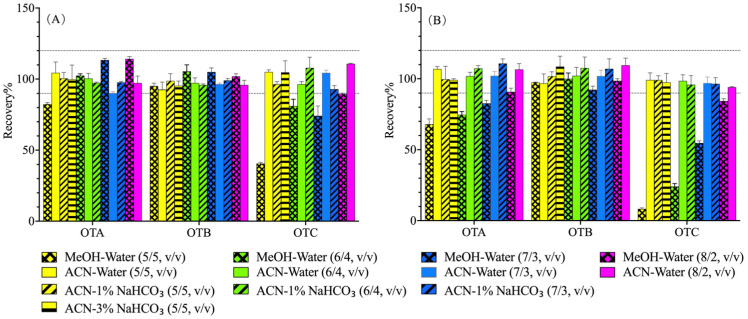
Extractive effect of ochratoxins in roasted coffee (**A**) and Sichuan pepper (**B**) matrix by different solvents.

Based on these stability findings, the extraction efficiencies of various ACN-water and MeOH-water systems were systematically compared in complex matrices (roasted coffee and Sichuan pepper). ACN-based systems (50–80%, *v*/*v*) consistently yielded high and stable extraction efficiencies (>90%) for all three analytes (Figure 2). In contrast, MeOH-based systems showed markedly lower efficiencies for OTC, particularly in the pepper matrix.

**Figure 3:**
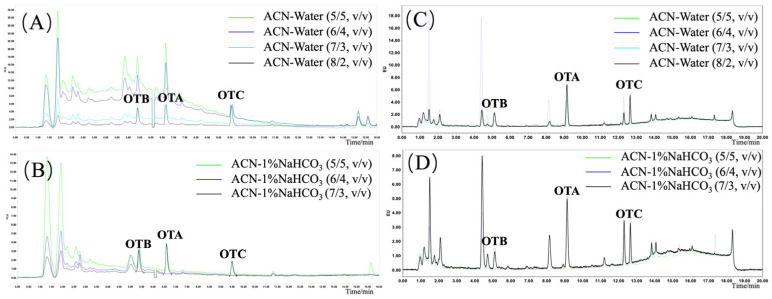
Liquid chromatographs of ochratoxins in roasted coffee (**A**,**B**) and Sichuan pepper (**C**,**D**) matrix by different extraction solvents. ((**A**,**C**): extracted by ACN-water; (**B**,**D**): extracted by ACN- NaHCO_3_ solvent).

Although efficiencies were comparable across different ACN-water ratios, the ACN-water (8/2, *v*/*v*) solvent was selected as optimal. This system provided a superior clean-up effect, evidenced by a cleaner baseline and reduced matrix interference in chromatograms, especially for coffee (Figure 3A,B). This is attributable to the more effective precipitation of proteins and other endogenous interferents at higher organic solvent concentrations.

### 3.2. Optimization of IAC Condition and Clean-Up Protocols

With the purpose of establishing a reliable method for the simultaneous determination of ochratoxin A, B, and C in complex matrices, the performance of modern IACs and the clean-up protocol were systematically evaluated. Firstly, OTA immunoaffinity columns from nine mainstream brands, both domestic and international, were collected. 0 mL and 1 mL of a mixed standard solution containing 100 ng/mL OTA, OTB, and OTC in PBS solvent were added, respectively, followed by direct loading, elution, detection using HPLC-FLD, and calculation of the absolute recovery rate. This study demonstrated that several commercial IAC brands exhibit high cross-reactivity (>85%) for both OTB and OTC, with no background interference, while existing methods target only OTA (Table 2). This finding is critical, as it addresses a key limitation of existing methods and commercial IACs, which are predominantly designed for OTA alone due to historical constraints in antibody specificity and standard availability. This high cross-reactivity, combined with the optimized ACN-water extraction solvent that ensures the stability of all three analogues, establishes a validated and reliable foundation for the simultaneous quantification of OTA, OTB, and OTC from complex food matrices.

**Figure 4:**
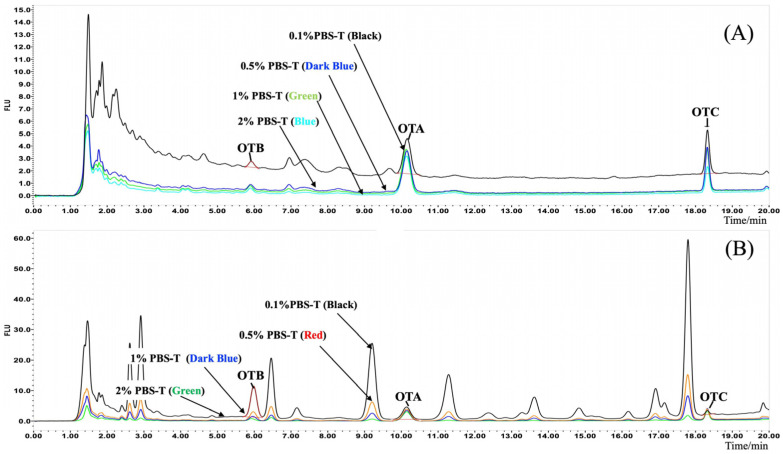
Chromatographic baseline and efficacy of roasted coffee (**A**) and Sichuan pepper (**B**) matrix extracted supernatant diluted with different PBS-T buffer.

To optimize the IAC purification step, the loading solution composition was first investigated. Dilution of the ACN-water (8/2, *v*/*v*) extract with conventional PBS buffer, a step required to reduce the ACN concentration below the column’s tolerance threshold (typically <8%), resulted in significant turbidity and column clogging when processing complex matrices like roasted coffee and Sichuan pepper. To address this, the efficacy of adding Tween-20 to the PBS dilution buffer was evaluated as a solubilizing agent. The addition of Tween-20 visibly reduced sample turbidity and the adhesion of coloured substances to the IAC packing material, leading to cleaner chromatograms and lower baseline noise (Figure 4).

**Figure 5:**
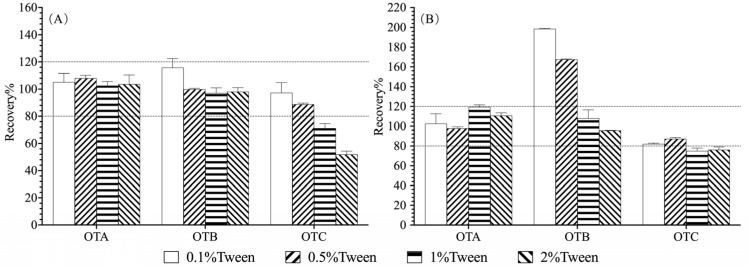
Recoveries of ochratoxins in roasted coffee (**A**) and Sichuan pepper (**B**) matrix extracted supernatant diluted with different PBS-T buffer.

However, the concentration of Tween-20 was found to significantly impact analyte recoveries (Figure 5). While OTA recovery remained high (>90%) across all tested concentrations, OTC recovery was adversely affected at higher surfactant levels, declining to <80% at Tween-20 concentrations of 1% and 2%. Conversely, in the pepper matrix, 0.1% and 0.5% Tween-20 in PBS solutions resulted in apparent OTB recoveries exceeding 150%. This was subsequently confirmed by UHPLC-MS/MS to be caused by co-eluting matrix interference, indicating incomplete purification. Based on these findings, 0.5% PBS-T was selected as the optimal dilution buffer, as it maintained high OTC recovery (>80%) while effectively solubilizing the matrix components and minimizing OTB interference in the coffee matrix.

**Figure 6:**
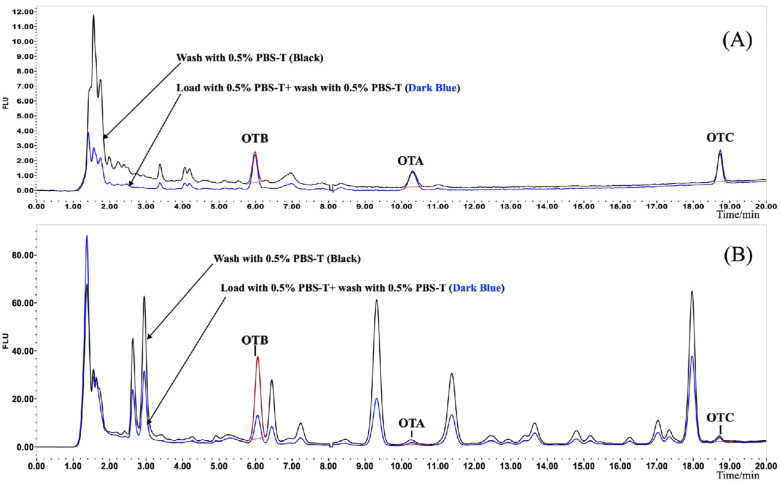
Liquid chromatographs and efficacy of ochratoxins in roasted coffee (**A**) and Sichuan pepper (**B**) matrix after PBS-T buffer washing.

To resolve the significant OTB interference observed with the 0.5% PBS-T loading buffer, the washing protocol was subsequently optimized. A water wash was first assessed but was found to slightly decrease recoveries for all three analytes; it was therefore excluded to ensure method robustness and reproducibility. A modified protocol was then tested, introducing an additional 10 mL wash with 0.5% PBS-T prior to the standard 10 mL PBS wash. This additional step successfully eliminated the OTB interference in the pepper matrix (reducing recoveries to a quantitative range of 100–118%) without compromising OTA or OTC recoveries. This modification also visibly improved the cleanliness of the final eluate (Figure 6). The final optimized washing procedure was thus established as 10 mL of 0.5% PBS-T followed by 10 mL of PBS.

### 3.3. Validation of HPLC-FLD and UHPLC-MS/MS Methodology

Following the optimization of the complete IAC protocol, the method was validated for both HPLC-FLD and UHPLC-MS/MS detection, utilizing roasted coffee and Sichuan pepper as the two representative matrix samples.

For all the calibration curves, quadratic correlation coefficients (*r*) above 0.999 were achieved for OTA, OTB, and OTC with both systems within their respective calibration ranges. The LC-MS/MS method demonstrated superior sensitivity, achieving LOQs of 0.1 µg/kg for all analytes, which was lower than the HPLC-FLD method (0.2–0.3 µg/kg), as shown in Table 3. LOQs were much lower than the minimums MRL established in coffee and spices (i.e., 3 μg/kg and 15 μg/kg for OTA) [16,17], according to Commission Regulation (EC) No 915/2023 and GB 2761-2017.

Accuracy and precision were confirmed in roasted coffee and Sichuan pepper. For both detection methods, mean recoveries (82.00–112.51%) and precision (RSD < 8.81%) at three spike levels (0.3, 5, 10 µg/kg) were well within accepted analytical criteria. These values are in accordance with Commission Regulation (EC) No. 401/2006 [32], which establishes minimal recoveries between 70 and 110% for >1 µg/kg spiked samples and 50–120% recoveries for <1 µg/kg spiked samples.

Matrix effects (ME) for the UHPLC-MS/MS method revealed significant analyte- and matrix-dependent signal suppression or enhancement, categorized into 3 types, namely, soft (±20%), medium (±30%), and strong ME (±50%) [33]. In this work, the matrix effect was examined for both coffee and spice categories by five different samples. As shown in Table 4, for ^13^C-OTB, the effect was consistently mild across all five matrices, with all values falling within the ±20% range (93.63–108.0%). ^13^C-OTA also demonstrated predominantly mild suppression or enhancement (82.49–109.9%), except for the induced medium signal suppression (72.65%) in white pepper. In stark contrast, ^13^C-OTC exhibited significant signal suppression in all tested matrices. It is noticeable that strong suppression (below 50%) appeared in roasted coffee (35.27%) and was particularly severe in the white pepper matrix (12.61%). The results demonstrate a clear compound- and matrix-dependent pattern. The pronounced and variable suppression, especially for ^13^C-OTC, confirms the necessity of using isotope-labelled internal standards to compensate for these effects and ensure accurate quantification.

### 3.4. Comparison of HPLC-FLD and UHPLC-MS/MS Method

The method’s reliability in real-world applications was further verified using FAPAS reference materials and a proficiency test. OTA results for a coffee reference material (FAPAS 17232) and a composite spice proficiency testing (PT) sample were within the assigned value ranges (Table 5). Notably, the *z*-score for the composite spice sample was 1.6, indicating satisfactory performance and comparability with other international laboratories. A statistical (ANOVA) comparison of OTA results obtained by HPLC-FLD and UHPLC-MS/MS revealed no significant difference (*p* > 0.05), confirming that both detectors provide equivalent and accurate quantification when coupled with the optimized IAC clean-up.

### 3.5. Comparing the Method with Previous Studies

The number of existing methods and standard analytical methods focus narrowly on OTA analysis, overlooking the cumulative risk of OTB and OTC (as listed in Table 6). Contrastingly, our single, unified workflow provides simultaneous and validated determination of all three analogues. This method’s key advantage lies in its robust applicability across diverse, highly complex food matrices (e.g., roasted coffee, multiple spices), surpassing the single-matrix focus of many reports. This robustness is achieved through the stable ACN-water extraction (preventing OTC degradation) and the novel Tween-20 IAC cleanup. Furthermore, our UHPLC-MS/MS platform achieves superior sensitivity, with an LOQ of 0.1 g/kg for all analogues, making this method simpler, more comprehensive, and substantially more sensitive than previous approaches for multi-OT monitoring.

### 3.6. Identification and Quantification of OTs in Commercial Coffee and Spices

Finally, in terms of highly sensitive and efficient detection, the optimized and validated IAC-UHPLC-MS/MS method was utilized to determine the occurrence of OTs in commercial coffee and spice samples. A total of 40 samples, including 19 coffee samples (instant, roasted, and black) and 21 spice samples (Sichuan pepper, cumin, and white pepper), were collected from local supermarkets and online shops. The measured values for the OTs in these samples are summarized in Table S2. Overall, OT contamination was observed in 19 out of 40 samples (47.5%), demonstrating the persistent exposure risk in these commodities. Importantly, OTC was not detected in any of the analyzed matrices. OTA was the most frequently detected analogue, found in 10 out of 19 coffee samples and 9 out of 21 spice samples. In roasted coffee, OTA concentrations ranged from 0.42 µg/kg (S12) to 3.46 µg/kg (S7). Notably, this study revealed the co-occurrence of OTA and OTB in 3 out of 8 cumin samples. The concentrations of OTB were 0.20 µg/kg (S27) and 1.05 µg/kg (S29), with another sample (S31) showing detectable levels alongside OTA. The co-occurrence of OTA and its less-toxic analogue OTB has been previously reported in other food commodities [15,25,38]. While the UHPLC-MS/MS method achieved a low LOQ (0.1 μg/kg) and the optimized ACN-water extraction ensured OTC stability during analysis, the non-detection is likely due to the combination of its lower natural prevalence compared to OTA/OTB and its unstable nature (ethyl ester), which makes it prone to degradation in the food matrix over time (e.g., during storage or processing).

The OTA levels detected in the coffee and spice samples (up to 3.46 μg/kg) generally fall below the current maximum EC regulatory limits, suggesting a manageable immediate risk based on OTA alone. However, the confirmed co-occurrence of OTA and OTB (in 3 of 8 cumin samples) is a critical finding. Given that OTB is known to possess toxic effects and may exert additive or synergistic toxicity with OTA, its simultaneous detection, now enabled by this validated method, is crucial for moving towards a more accurate ‘Total Ochratoxin Load’ risk assessment. These data underscore the necessity for regulatory bodies to consider the cumulative risk posed by multiple OT analogues, rather than focusing solely on OTA. The proposed approach provides a robust and essential tool for generating comprehensive occurrence data, enabling more accurate and complete toxicological risk assessments for these complex matrices.

### 3.7. Adaptability of the Method to Other Matrices

The unified extraction and cleanup approach developed here provides a robust and adaptable framework for multi-OTs analysis in other high-risk food matrices, such as cereals, dried fruits, wine, and cocoa products. These matrices share common analytical challenges (e.g., high lipid content, intense pigmentation, complex extractable co-contaminants) that are effectively mitigated by the IAC purification enhanced with Tween-20. Pending specific single-matrix validation, the method holds strong potential as a versatile tool for comprehensive mycotoxin monitoring across diverse food categories.

## 4. Limitations

While this study successfully developed and validated a robust method for multi-OTs analysis, some limitations should be noted. Firstly, the analysis of commercial samples was limited to forty items, primarily collected from one region; a larger, multi-regional sampling would be required to generate data truly representative of national or provincial OTs prevalence and facilitate robust statistical analysis between different food groups. Secondly, while the ACN-water extraction system proved stable for OTC during the short-term analysis procedure, a long-term stability study of OTC in various raw and processed food matrices was not performed, which could provide further insight into the factors governing its prevalence.

## 5. Conclusions

This study successfully developed and comprehensively validated a robust, unified analytical method for the simultaneous determination of OTA, OTB, and OTC in coffee and spices, thereby addressing a critical analytical gap in multi-OT monitoring. The method’s reliability stems from key innovations that successfully address challenges of analyte instability and matrix complexity: (1) A stable acetonitrile-water (8/2, *v*/*v*) extraction system was established to prevent OTC degradation; (2) For the first time, high cross-reactivity (>85%) of commercial OTA-IACs toward OTB and OTC was demonstrated; (3). A novel IAC purification protocol, utilizing 0.5% Tween-20 in PBS, was developed to effectively eliminate severe matrix interference. The method was validated on both HPLC-FLD and UHPLC-MS/MS platforms, proving accurate and sensitive. Real sample analysis confirmed the co-occurrence of OTA/OTB, underscoring the necessity of this multi-component approach for comprehensive risk assessment. This validated methodology establishes an urgently needed, high-performance analytical tool. The next logical step is to apply this validated method to a larger, more diverse collection of samples from various geographical origins and food matrices (e.g., cereals, cocoa, dried fruit) to generate statistically representative and globally relevant occurrence data. These findings will provide reliable data for OTB and OTC—data that are currently scarce—thereby facilitating the future establishment of regulatory standards and informing public health policies. Ultimately, this method enables a critical shift in risk assessment toward a “Total Ochratoxin Load” approach and serves as a robust template for regulatory method development for these globally important food commodities.

## Figures and Tables

**Table 1:** Multiple reaction monitoring (MRM) parameters of ochratoxins.

Compound	Parent Ion/(m/z)	Product Ion/(m/z)	DP/V	CE/eV
OTA	404.0	239.1 *, 358.1	100	34, 20
OTB	370.0	205.1 *, 324.1	70	28, 19
OTC	432.1	239.1 *, 358.1	70	35, 22
^13^C-OTA	424.2	250.1	100	32
^13^C-OTB	390.2	216.1	70	33
^13^C-OTC	452.2	250.1	70	36

Note: * for quantitative ion.

**Table 2:** Column reactivity for OTs in different commercial IACs.

IAC Brand No. *^b^*	Column Reactivity % *^a^*		
	OTA	OTB	OTC
1 *^c^*	98.90	107.8	100.6
2	109.4	109.6	88.41
3	95.31	93.75	90.00
4	108.4	108.1	86.00
5	108.9	107.0	92.50
6	98.04	99.20	71.28
7	88.18	87.68	67.44
8	82.4	80.32	77.52
9	89.63	83.98	21.94

Note: *^a^*, column reactivity% = measured content/theoretical column-passing content × 100; theoretical column-passing content is 100 ng; *^b^*, IAC brand details were listed in Table S1; *^c^*, IACs used in this work.

**Table 3:** Validation parameters for ochratoxins by HPLC-FLD and LC-MS/MS methodology.

Compound	HPLC-FLD				UHPLC-MS/MS			
	LOQ (µg/kg)	Spiking (µg/kg)	Roasted Coffee *	Sichuan Pepper *	LOQ (µg/kg)	Spiking (µg/kg)	Roasted Coffee *	Sichuan Pepper *
OTA	0.3	0.3	98.99 ± 8.81	95.83 ± 5.35	0.1	0.3	95.93 ± 2.04	99.18 ± 4.57
		5	99.97 ± 4.68	105.63 ± 2.50		5	104.07 ± 5.12	99.11 ± 2.50
		10	96.58 ± 4.71	99.80 ± 4.41		10	109.83 ± 7.11	96.99 ± 1.96
OTB	0.2	0.3	85.56 ± 6.36	111.11 ± 5.59	0.1	0.3	92.26 ± 2.67	110.09 ± 3.94
		5	100.44 ± 3.48	92.41 ± 1.79		5	95.97 ± 4.06	95.49 ± 3.97
		10	89.68 ± 3.86	91.71 ± 3.11		10	100.53 ± 7.85	94.05 ± 2.84
OTC	0.2	0.3	86.11 ± 5.15	82.22 ± 5.54	0.1	0.3	103.94 ± 3.51	112.51 ± 3.55
		5	95.54 ± 1.99	82.17 ± 3.01		5	98.83 ± 4.86	99.89 ± 3.76
		10	85.69 ± 4.30	82.00 ± 2.10		10	104.05 ± 5.09	98.88 ± 1.55

Note: * average ± standard deviation, *n* = 6.

**Table 4:** Matrix effect for ochratoxins in coffee and spice matrices by UHPLC-MS/MS.

Compound	Instant Coffee	Roasted Coffee	Cumin	Sichuan Pepper	White Pepper
^13^C-OTA	107.7	88.32	109.9	82.49	72.65
^13^C-OTB	93.63	108.0	105.8	101.4	96.38
^13^C-OTC	74.53	35.27	52.87	62.21	12.61

**Table 5:** Detection results of OTA in quality samples by HPLC-FLD and UHPLC-MS/MS methods.

Samples	Assigned Content (µg/kg)	HPLC-FLD (µg/kg)	UHPLC-MS/MS (µg/kg)
Coffee	3.18 ± 1.4	3.54 ± 0.20 *	3.55 ± 0.24
Mixed spice	16.0 ± 7.0	20.1 ± 1.4	20.5 ± 0.8

Note: * average ± standard deviation, *n* = 6.

**Table 6:** Comparison of the proposed method with previously reported methods for the determination of OTs in coffee and spices.

Analyte.	Extraction	Pretreatment	Matrix	Instrument	LOD (μg/kg)	LOQ (μg/kg)	Reference
OTA/OTB/OTC	ACN-water (8/2, *v*/*v*)	IAC	Coffee and spices	LC-MS/MS HPLC-FLD	0.1; 0.3	0.3; 1	This study
OTA	MEOH-3%NaHCO_3_ (50 + 50, *v*/*v*)	IAC	spices, liquorice, cocoa, and cocoa products	HPLC-FLD	/	1	[30]
OTA	MEOH-3%NaHCO_3_ (50 + 50, *v*/*v*)	Phenyl silane SPE-IAC	Roasted coffee and barley	HPLC-FLD	/	0.6	[31]
OTA	MEOH-water (8/2, *v*/*v*)	IAC	Coffee	HPLC-FLD	0.47	1.23	[34]
AFTs/OTA	MEOH-ACN (6/4, *v*/*v*)	QuEChERS method	Raw coffee beans	LC-MS/MS	/	0.6	[35]
AFTs/OTA	3%NaHCO_3_; ACN-water; MEOH-0.5%NaHCO3	IAC	Spices	HPLC-FLD	<0.1	/	[36]
AFTs/OTA	MEOH-water (8/2, *v*/*v*)	IAC	Chilli powder	LC-MS/MS HPLC-FLD	/	1; 0.5	[37]

## Data Availability

The original contributions presented in this study are included in the article/Supplementary Material. Further inquiries can be directed to the corresponding author.

## Supplementary Materials

The following supporting information can be downloaded at: https://www.mdpi.com/article/10.3390/foods14234102/s1. Supplementary Tables: Supplementary Table S1, Detailed information of commercial OTA-Clean IACs; Supplementary Table S2, The measured values of the OTs in the 40 commercial samples obtained from the supermarkets and online shops in Zhejiang Province, measured by the UHPLC-MS/MS method assisted by an optimized IAC clean-up process. Figure S1: Workflow for OTs pretreatment and detect.
