# Supplementary material for: Comprehensive IAC Cross-Reactivity Validation and Stabilized Method Development for Ochratoxin A, B, and C in Complex Coffee and Spice Matrices
Source: Foods. 2025 Nov 28;14(23):4102. doi: 10.3390/foods14234102 (PMC12692647; doi:10.3390/foods14234102)
Supplement: Supplementary file 1 [file foods-14-04102-s001.zip › foods-3995248-supplementary.pdf]

Figure S1 Workflow for OTs pretreatment and detect.

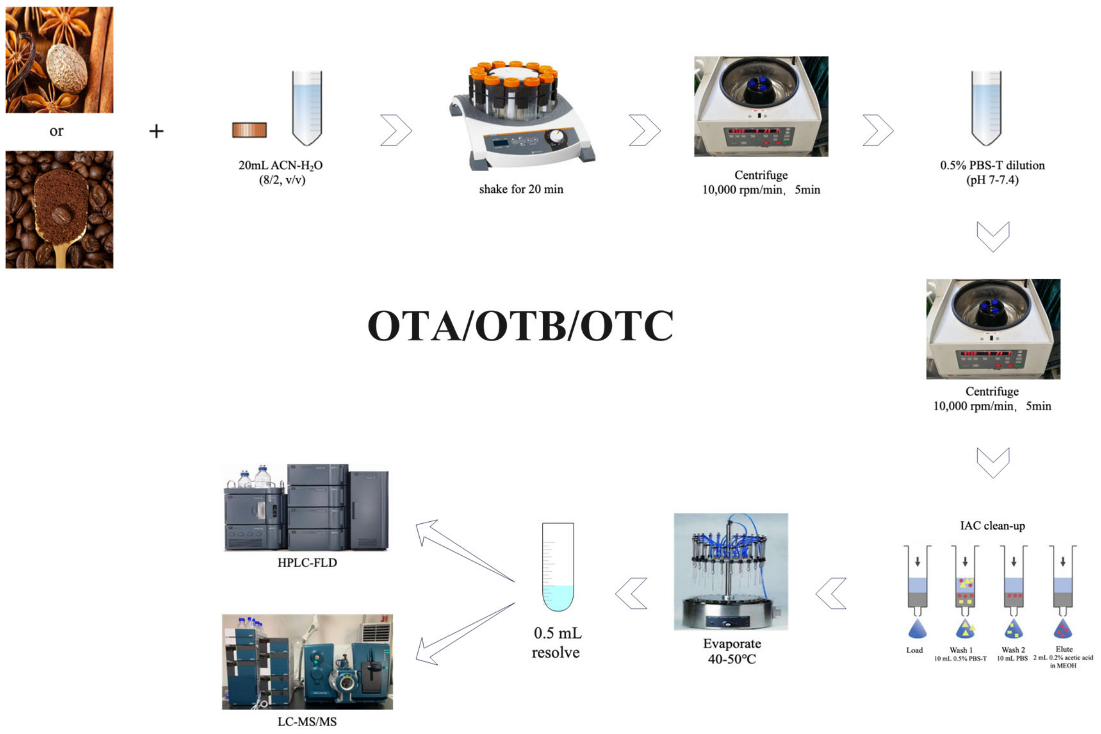

Table S1. Detail information of commercial OTA-Clean IACs

No.	Brand	Capacity (ng)	Supplier	Country
1	HCM OTA-IAC	200	Meizheng Bio-tech Co., Ltd	China
2	PriboFast®	200	Pribolab Pte. Ltd.	China
3	CLOVER IAC-SEP@	100	Beijing ClovertechLimited Company	China
4	OchraStar@	500	Romer Labs Division Holding GmbH	Austria
5	OCHRAPREP®	100	R-Biopharm AG	Germany
6	Welchrom@AO	100	Welch technology (Shanghai) Co., Ltd	China
7	Casco@ OTA-IAC	100	Casco Biotech Co., Ltd	China
8	Suwei OTA-IAC	100	Jiangsu Suwei Microorganism Research Co., Ltd	China
9	CNW@	100	ANPEL Laboratory Technologies (Shanghai) Inc.	China

Table S2. The measured values of the OTs in the 40 commercial samples obtained from the supermarkets and online-shops in Zhejiang Province, measured by UHPLC-MS/MS method assisted by an optimized IAC clean-up process.

Sample No.	Sample type	Measured values for the OTs (μg/kg)		
		OTA	OTB	OTC
S1	Instant Coffee	-	-	-
S2	Instant Coffee	-	-	-
S3	Instant Coffee	-	-	-
S4	Instant Coffee	-	-	-
S5	Instant Coffee	-	-	-
S6	Instant Coffee	-	-	-
S7	Roasted Coffee	3.46	-	-
S8	Roasted Coffee	0.43	-	-
S9	Roasted Coffee	0.52	-	-
S10	Roasted Coffee	1.66	-	-
S11	Roasted Coffee	-	-	-
S12	Roasted Coffee	0.42	-	-
S13	Roasted Coffee	-	-	-
S14	Roasted Coffee	1.07	-	-
S15	Roasted Coffee	0.66	-	-
S16	Black Coffee	1.92	-	-
S17	Black Coffee	1.44	-	-
S18	Black Coffee	-	-	-
S19	Black Coffee	0.72	-	-
S20	Sichuan Pepper	-	-	-
S21	Sichuan Pepper	-	-	-
S22	Sichuan Pepper	-	-	-
S23	Sichuan Pepper	-	-	-
S24	Sichuan Pepper	D.	-	-
S25	Sichuan Pepper	-	-	-
S26	Cumin	-	-	-
S27	Cumin	0.32	0.20	-
S28	Cumin	-	-	-
S29	Cumin	0.53	1.05	-
S30	Cumin	-	-	-
S31	Cumin	0.42	D.	-
S32	Cumin	-	-	-
S33	Cumin	D.	-	-
S34	White Pepper	-	-	-
S35	White Pepper	D.	-	-
S36	White Pepper	-	-	-
S37	White Pepper	0.48	-	-
S38	White Pepper	-	-	-
S39	White Pepper	0.69	-	-
S40	White Pepper	0.19	-	-

"D.": detectable; "-": no signal
